# Google Search Trends in Oncology and the Impact of Celebrity Cancer Awareness

**DOI:** 10.7759/cureus.5360

**Published:** 2019-08-10

**Authors:** Tasneem Kaleem, Timothy D Malouff, William C Stross, Mark R Waddle, Daniel H Miller, Audrey L Seymour, Nicholas G Zaorsky, Robert C Miller, Daniel M Trifiletti, Laura Vallow

**Affiliations:** 1 Department of Radiation Oncology, Mayo Clinic Florida, Jacksonville, USA; 2 Department of Radiation Oncology, Gamma West Cancer Services, Idaho Falls, USA; 3 Department of Public Affairs, Mayo Clinic Florida, Jacksonville, USA; 4 Department of Radiation Oncology, Penn State University, Hershey, USA; 5 Department of Radiation Oncology, University of Maryland, Baltimore, USA

**Keywords:** google, search engine, cancer, celebrity

## Abstract

Introduction

There is widespread public interest when celebrities are diagnosed with cancer. We sought to assess how this interest impacts awareness of prevalent cancers.

Methods

We reviewed common cancer-related search terms using Google Trends (Google LLC, Mountain View, CA) between the years 2004 and 2017 and retrospectively correlated these findings with media or celebrity-related events. The Google Trends application was used to obtain the “search volume index” (SVI), defined as the number of searches for a specific term standardized to the total number of searches over that time period. Data were presented in a graphical format. Isolated peaks of greater than 25% from the baseline SVI were identified. Using the date of the peaks, a further search was performed to determine if any event in the media triggered the peak.

Results

“Lung Cancer,” “Pancreas Cancer,” “Endometrial Cancer,” “Cervical Cancer,” “Brain Cancer,” and “Glioblastoma” each had the highest peak correspond with a celebrity-related event covered in the media. These search terms displayed several additional isolated peaks, the majority of which could all be correlated with a significant media event (%). The search term “Breast Cancer” consistently had a peaked interest during October (breast cancer awareness month). Breast cancer events relating to public figures had little to no relative impact on search volume during this period. None of the other cancer search terms displayed the same cyclical pattern during their respective awareness months. Colon, rectal, and prostate cancer demonstrated stable search volumes over time, without an isolated peak.

Conclusion

Internet search activity among English speakers of most general cancer terms exhibit peaks coinciding with events that occur to celebrity figures or advances in medicines that are substantially covered in the media. In all cases but “breast cancer,” these events lend to higher search activity as compared to campaigns and awareness months. Our study suggests that media coverage of public figures with cancer may trigger substantial Internet interest in non-breast cancers, more so than traditional efforts to raise awareness.

## Introduction

Assessing the role of the media and celebrity cancer-related events can be useful in understanding what triggers public awareness and interest in various cancers. News and announcements related to famous figures in the media have historically shown an impact in patient understanding, awareness, as well as referral patterns. A well-known example is the public announcement of Angelina Jolie undergoing a bilateral mastectomy due to a family history of cancer and carrying a mutation of the BRCA1 gene [1]. The “Angelina Jolie effect” had a significant influence on the public regarding the understanding and perception of breast cancer, mastectomy, and the BRCA1 gene [1]. The extensive media coverage also translated to higher referrals and inquiries. In a family cancer center in Canada, a retrospective analysis noted that the number of women referred for genetic counseling increased by 90% after six months and remained high one year after Angelina Jolie’s story, with an increase of 88% from baseline [2]. One of the most accessible media outlets of celebrity announcements is the Internet.

In general, Internet utilization and cancer searches over time have increased. Health Information National Trends Survey (HINTS) data 2002-2014 show a rapid increase in Internet usage for the general public, from 63% of adults to 83% in 2014. Among those who had actively searched for information on cancer in 2003, approximately 48% indicated that they had used the Internet first; only 7% had gone to their providers. Both of these percentages increased by the year 2008, with 55% searching the Internet first and 23% reportedly relying on their doctors as the first port of call [3].

With Internet usage on the rise, search engines become a ubiquitous vehicle for obtaining information. Among the search engine landscape, Google (Mountain View, CA, USA) is by far the most utilized. As evidence, in the month of December 2012, Google was used for 114.7 billion searches, which is 65.2% of the share of total searches on the Internet [4]. Google has seeped into our vernacular and has commonly become our first source for searching for information. Thus, search activity could be considered a measurable means to evaluate interest and awareness. These endpoints can be temporally evaluated using Google Trends in near-real-time, a freely available web tool that shows how often a particular search term is entered relative to the total search volume geographically and over time.

In an effort to evaluate the Internet in the world’s most common cancers, we reviewed common cancer-related search terms using Google Trends and determined whether high-interest periods correlated with media or celebrity-related events.

## Materials and methods

The Google Trends application was the main analytical tool utilized. Data from the application are provided as the “search volume index” (SVI), which shows the number of searches for a specific term per time point in relation to the total number of searches on the Google search engine during that time period. This is scaled from 0 to 100, 100 signifying the peak search volume for the search term during the time period.

The application was accessed for the current study on November 14, 2017. A separate search was performed for the following terms: “Breast Cancer,” “Mastectomy,” “BRCA1,” “Lung Cancer,” “Colon Cancer,” “Rectal Cancer,” “Pancreatic Cancer,” “Brain Cancer,” “Glioblastoma,” "Prostate Cancer," “Cervical Cancer,” and “Endometrial Cancer.” These terms were chosen to reflect the most common and deadly cancers worldwide, as well as search term availability within the range of topics in the current version of Google Trends [5]. We elected to include “Mastectomy” and “BRCA1” along with “Breast Cancer” and “Glioblastoma” with “Brain Cancer” as additional search terms, given the subjective popularity of those terms in the media at the time the study was performed. Data were searched worldwide from the date of first available data within Google Trends (January 1, 2004) to the current date (November 14, 2017).

From the resultant data, "isolated peaks" were defined as a greater than 25% spike above the baseline SVI. Using the date of the isolated peaks, further searches using site-specific search terms was performed to determine if any event immediately preceding the isolated peak was reported in the media. Special attention was paid to announcements, publications, deaths, interviews, or campaigns within the month preceding the peak (i.e. searches focused on events in September and October for a peak in October). Peaks in the SVI that correlated with a media event were recorded. Graphical representation was taken from the Google Trends application and correlated media events were plotted to indicate plausible relationships.

## Results

Breast cancer

**Figure 1:**
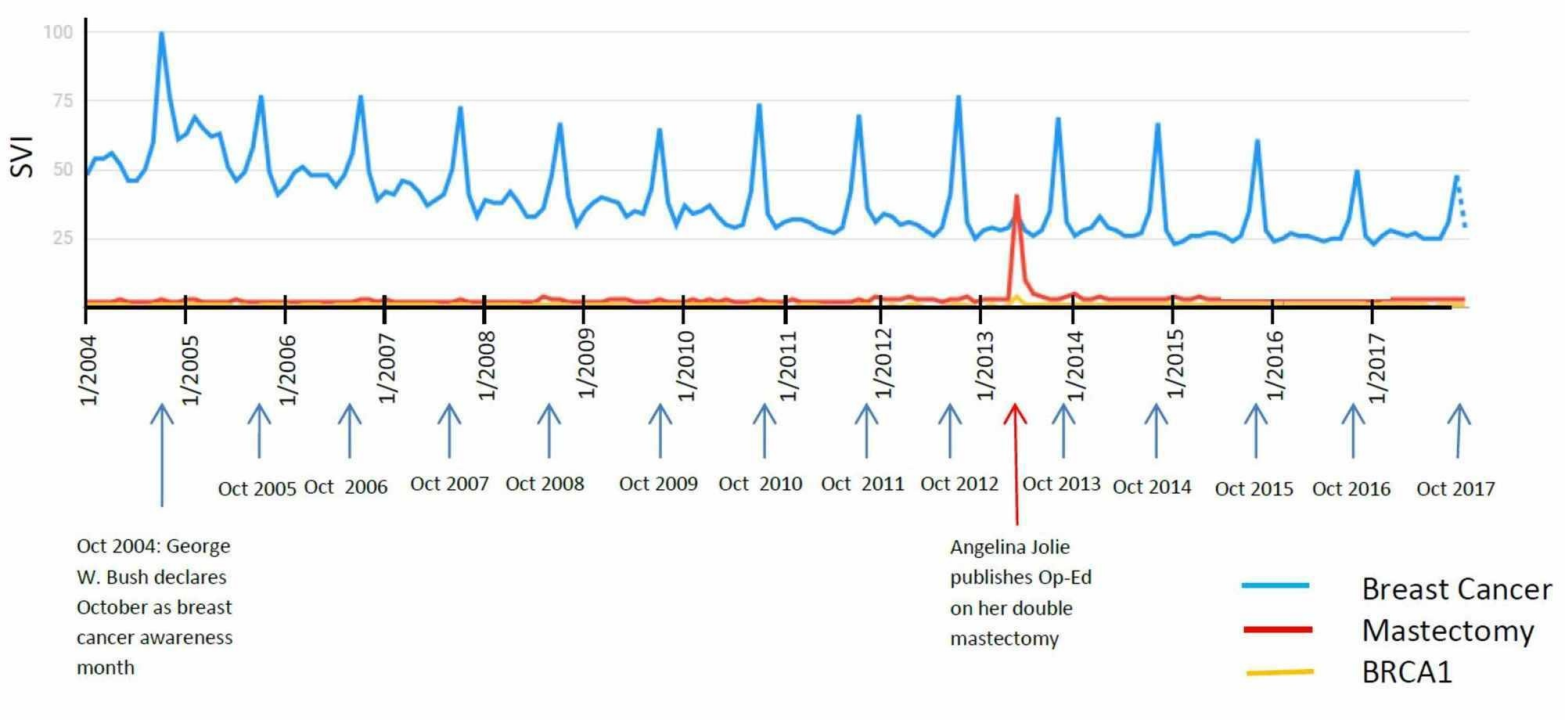
“Breast Cancer,” “Mastectomy,” and “BRCA1” search term results

In October 2004, President George W. Bush proclaimed October as breast cancer awareness month [6]. For the search term “Breast Cancer,” this timing correlated to the largest SVI peak at 100%, and, subsequently, every October onward, an isolated peak was displayed. The height of the isolated peak varied over time; however, a significant peak is always present (over 25% of the baseline). There were several celebrity announcements of breast cancer, including Julia Louis Dreyfus (9/28/2017), Christina Applegate (8/19/2008), Judy Blume (9/5/2012), and Newscaster Robin Roberts (9/19/2007). However, none of these announcements correlated with an isolated peak. Angelina Jolie announced her prophylactic mastectomy and BRCA1 genetic susceptibility in a New York Times Op-Ed [7]. Subsequently, in the same week, a significant peak was reported for the search term “Mastectomy,” which is the sole peak throughout the search term history (Figure 1). None of the isolated peaks in SVI of the term “Breast Cancer” correlated with a conventional celebrity announcement (0 of 14 isolated peaks, 0%).

Lung cancer

**Figure 2:**
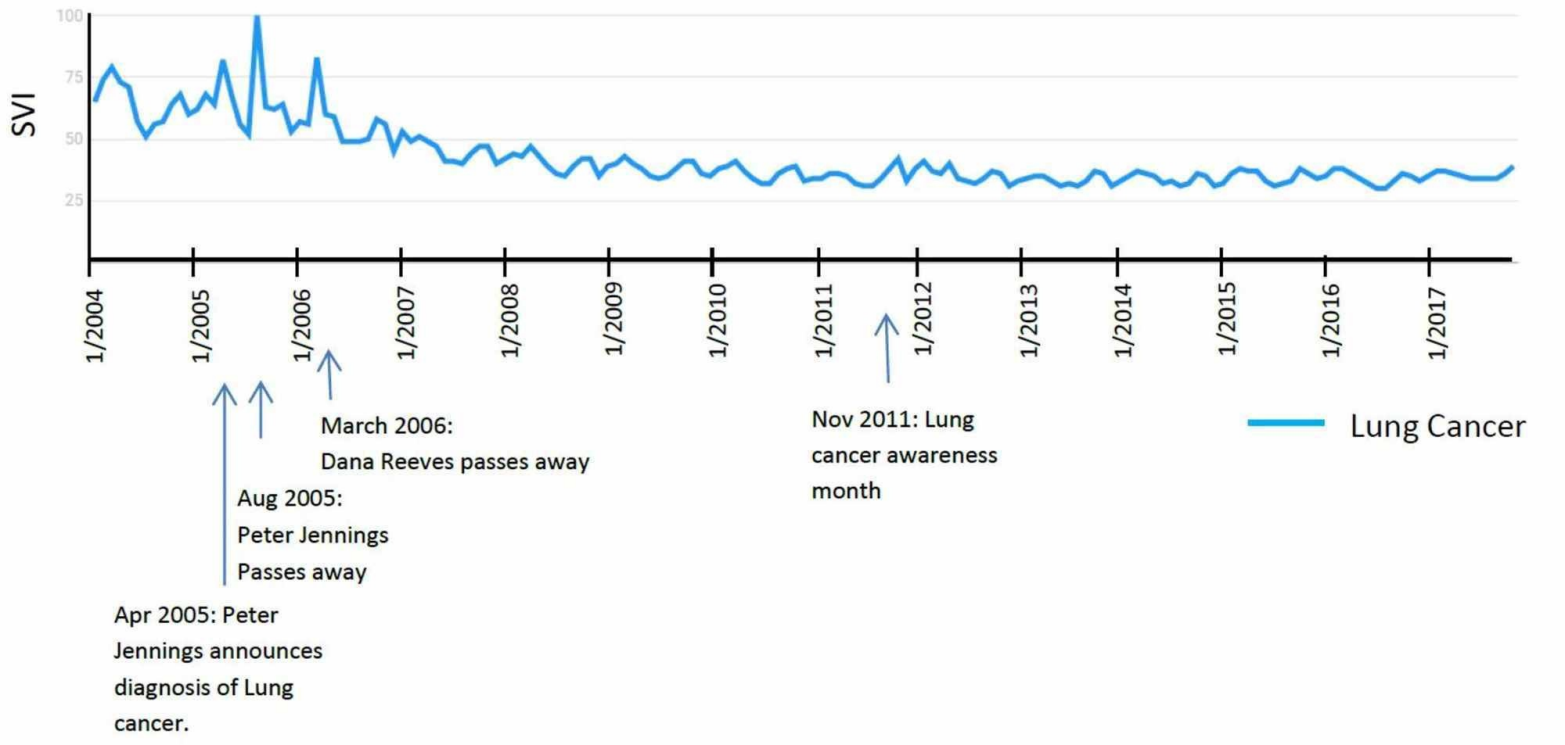
“Lung Cancer” search term results

Results for search term “Lung Cancer” are depicted in Figure 2. Three of the four isolated peaks in SVI of the term “Lung Cancer” correlated with a conventional celebrity announcement (three of four isolated peaks, 75%, Figure 2). The majority of the SVI peaks for lung cancer are reported before 2007, and they have gradually decreased over time. The highest peak is in August 2005, which correlated with the passing away of Peter Jennings [8]. Furthermore, his live announcement on ABC nightly news correlated with another peak in April 2005. The next highest peak is in March 2006, which correlates with the passing away of actress and activist Dana Reeves [9]. Another isolated peak was found in March 2004; however, this did not correlate with any announcement in the media. There have been no isolated peaks since 2006. Of note, November is lung cancer awareness month, and no peaks correlated temporally to November.

Colon and rectal cancer

**Figure 3:**
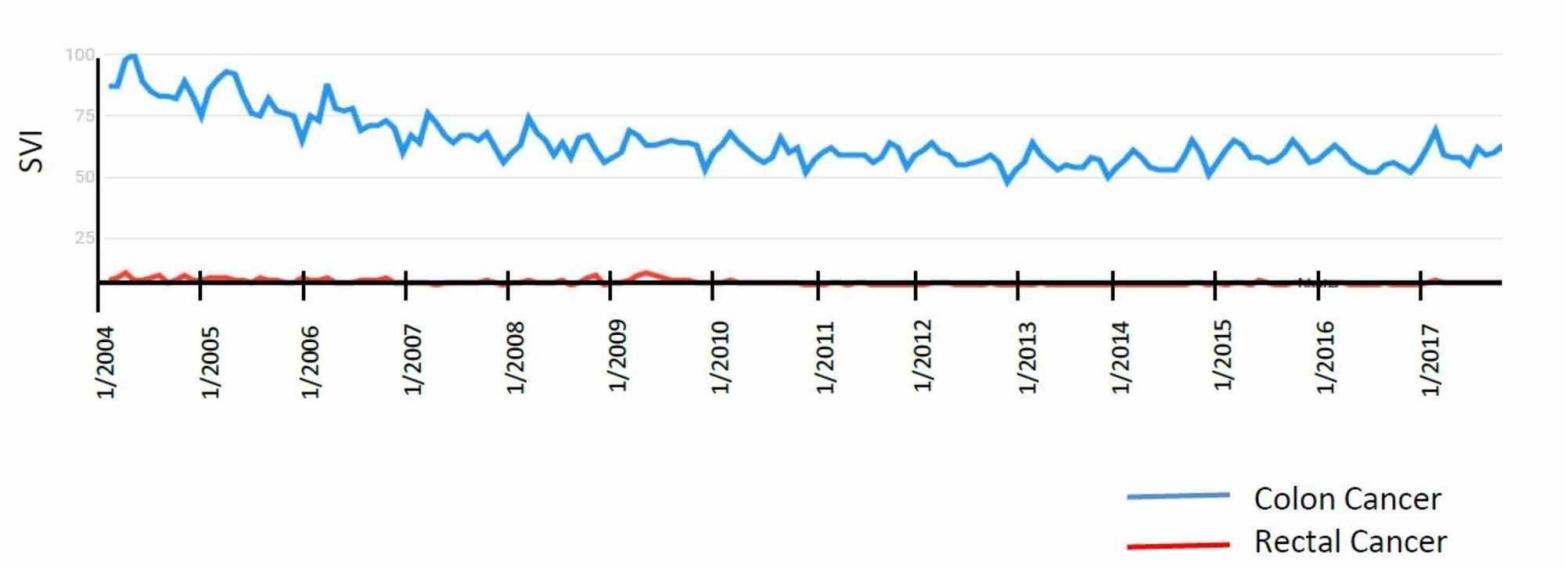
"Colon Cancer" and "Rectal Cancer" search term results

“Colon Cancer” and “Rectal Cancer” were both queried; however, neither search term showed isolated peaks, as defined. Colorectal cancer awareness month is in March, and it did not correlate with significant peaks. This was dedicated in February 2000 by President Bill Clinton (Figure 3) [10].

Prostate cancer

**Figure 4:**
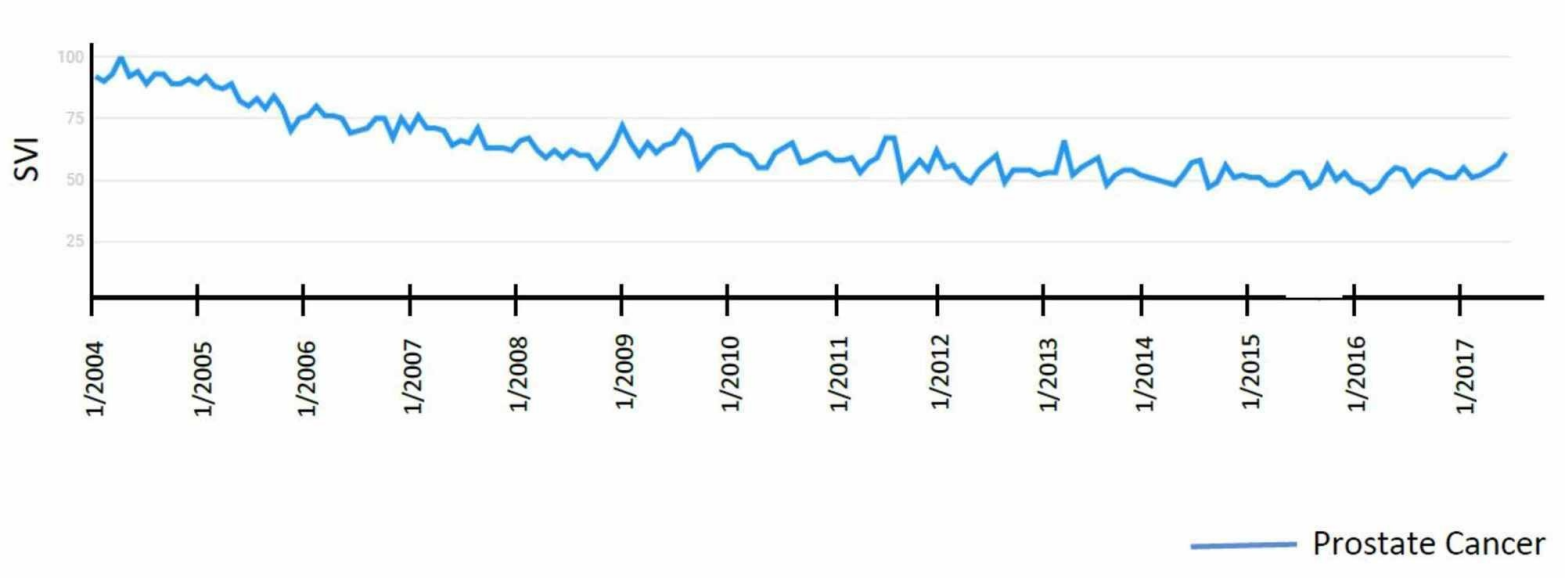
"Prostate Cancer" search term results

Prostate cancer has been present in the media through the announcements of many public figures. For example, Ben Stiller published an Op-Ed on October 4, 2016, discussing his experience with the diagnosis and treatments [11]. Other famous figures also announced their diagnosis, including Phil Lesh and Dr. Drew Pinskey. However, these announcements did not show any correlations, in fact, there were no significant peaks since 2004 (Figure 4). September 17th to September 24th is Prostate Cancer awareness week, and no isolated peaks were correlated around this time period.

Pancreatic cancer

**Figure 5:**
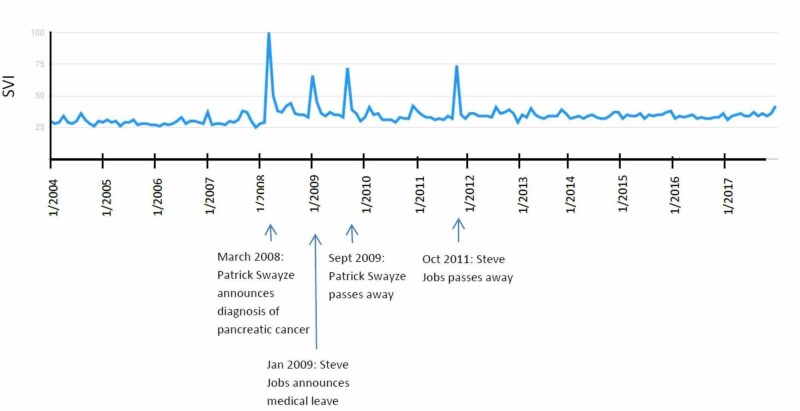
“Pancreatic Cancer” search term results

The “Pancreatic cancer” search term was queried and showed four significant peaks; all correlated with events in the media (four of four, 100%). The highest peak was in March 2008, which correlated with the announcement of the diagnosis of pancreatic cancer by actor Patrick Swayze. In January 2009 [12], another peak correlated with the media coverage surrounding Steve Jobs taking medical leave from Apple due to pancreatic cancer [13]. The next two peaks, in September 2009 and October 2011, correlate with the passing away of Patrick Swayze (2009) [14] and Steve Jobs (2011; Figure 5).

Endometrial cancer

**Figure 6:**
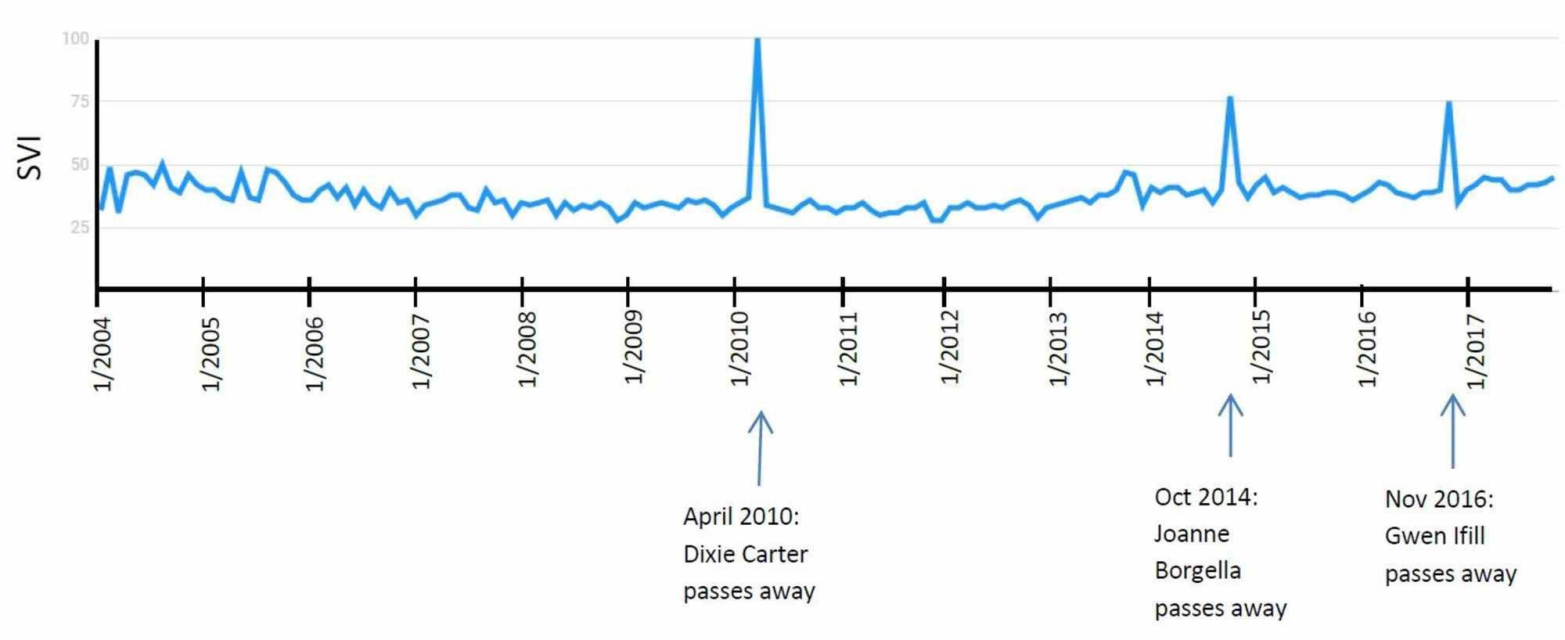
"Endometrial Cancer" search term results

The graphical representation of SVI for the search term “Endometrial Cancer” showed three significant peaks. All three peaks correlated with news in the media about a known figure (three of three, 100%). In April 2010, actress Dixie Carter passed away [15], which subsequently showed the highest SVI peak. The next peak, in October 2014, correlates with the passing away of Joanne Borgella, an American singer [16]. The last peak, in November 2016, correlates with the death announcement of Gwen Ifill, a popular journalist (Figure 6) [17]. Endometrial cancer awareness month is September, and it showed no significant activity over time.

Cervical cancer

**Figure 7:**
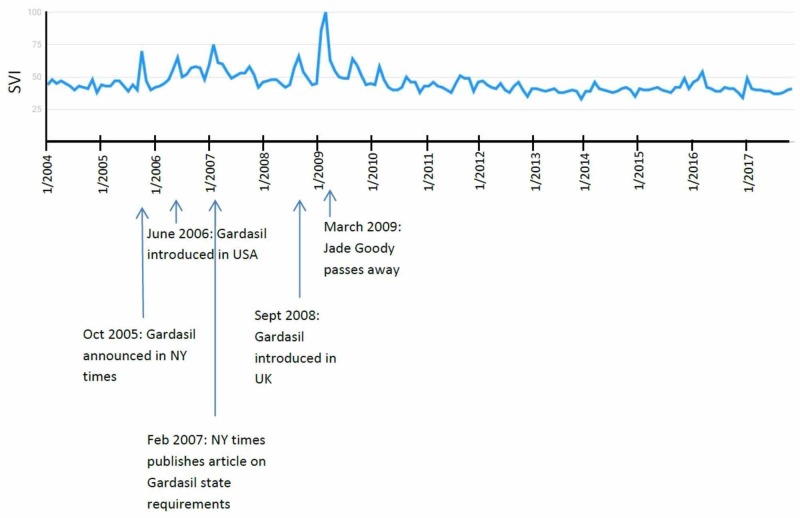
"Cervical Cancer" search term results

The “Cervical Cancer” SVI graph showed several isolated peaks, each of which correlated with a media announcement (five of five, 100%). The highest peak is in March 2009, when Jade Goody passed away at the age of 27 [18]. There are several peaks neighboring this peak, most of them coinciding with announcements of human papillomavirus (HPV) vaccination for cervical cancer. An article was published in the New York Times in October 2005 on the topic [19], and the vaccine was subsequently introduced to the United States in June 2006 and to the United Kingdom in September 2008. Each of these events has correlated peaks. There are several other isolated peaks, including February 2007 and September 2009, which coincide with many articles published on the ongoing debate of the Gardasil vaccine as a state requirement. Since 2009, however, there has been minimal significant activity of the search term “Cervical Cancer” (Figure 7).

Brain cancer and glioblastoma

**Figure 8:**
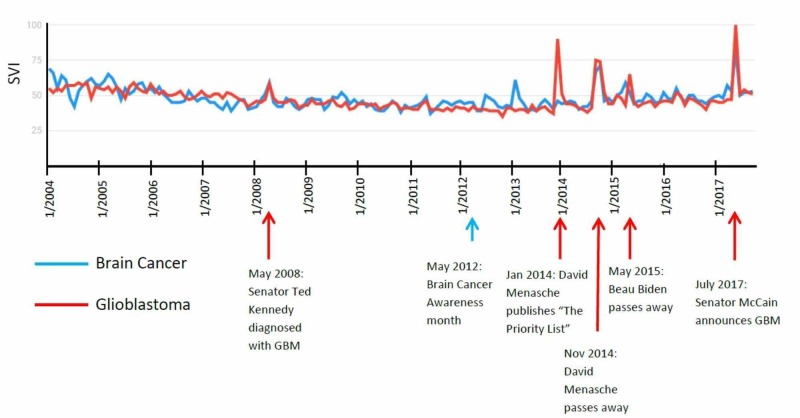
“Brain Cancer” and “Glioblastoma” search term results

Both “Brain Cancer” and “Glioblastoma” were used as search terms. Both curves showed parallel trends and peaks. Five of the six isolated peaks correlated with a media event (83%). The first isolated peak presented in May 2008, when the media covered Senator Ted Kennedy’s diagnosis of glioblastoma [20]. The next peak for “Glioblastoma” alone was in January 2014, when teacher David Menasche published “The Priority List” and received significant media attention regarding his diagnosis and novel [21]. He subsequently passed away in November 2014, which coincided with peaks for both “Brain Cancer” and “Glioblastoma.” Both terms peaked again in May 2015 after the announcement of Beau Biden passing away [22]. The highest peak for both search terms was in July 2017, when Senator John McCain announced his diagnosis of glioblastoma. Brain cancer awareness month is in May, and there were no correlated peaks except for the months that coincided with the passing away of Beau Biden and the diagnosis of Senator Ted Kennedy (Figure 8).

## Discussion

The aim of this study was to evaluate the impact of the media and events related to famous figures on general cancer search terms. Our study shows that “Breast Cancer” has a consistent interest during October, which is breast cancer awareness month. Events relating to public figures had little to no impact on breast cancer search volume. This is likely due to the prevalence of breast cancer, in which a high frequency of baseline searches can mask transient increases in search volumes. Interestingly, no other cancer site has the same, consistent pattern of isolated peaks during their respective awareness months or even isolated peaks at the initial awareness month announcement.

“Lung Cancer,” “Pancreas Cancer,” “Endometrial Cancer,” “Cervical Cancer,” and “Brain Cancer” and ”Glioblastoma” all had the highest peak coincide with a celebrity-related event covered in the media. These results show that public interest in non-breast cancers are most impactfully triggered by announcements in the lay media, particularly by celebrities.

Cancer awareness months are campaigns with the purpose of increasing interest, raising funds for research into the cause of cancer and its prevention, early diagnosis, treatment, and cure. Most importantly, they serve to educate the public on the importance of screening and early intervention. Previous comparisons have shown consistently higher search activity for breast cancer in October vs. lung or prostate cancer in their specific awareness months [23]. This correlates well with our current results. One possible explanation for the results is the higher relative prevalence of breast cancer, although similar trends were not found for prostate cancer [24]. Another explanation is that the strategy utilized by breast cancer campaigns gain more publicity. For example, National Football League (NFL) games sporting pink or iconic buildings, such as the White House and Empire State Building, lit in pink are strategies not readily used in other cancer campaigns and may serve to provide a sort of "annual celebrity media announcement" promoting breast cancer awareness. Another possibility is that women and higher socioeconomic demographic populations tend to utilize the Internet more [25]; they also tend to be a majority of the breast cancer population [24].

Colon, rectal, and prostate cancer did not show any significant peaks. Reasons include the paucity of famous public figures announcing the diagnosis of their disease. There have not been a concordant significant number of public figures given the frequency of these cancers (as compared to breast cancer) that have come forward discussing the experience with these disease sites. Furthermore, there has not been a new advancement that has brewed an ethical discussion, as exemplified by the HPV vaccine for cervical cancer. A recent paper reported on the content analysis of 344 articles published between January 2005 and December 2008 in 15 UK newspapers regarding HPV vaccination, discussing the positive as well as negative coverage [26]. This bipolar coverage most likely lent to the spawning of several peaks in Google activity over several years for “Cervical Cancer.” This type of coverage has never been present in the realm of colon, rectal, or prostate.

The effect of media coverage on the diagnosis and death of a famous figure due to cancer has been previously studied in the United States. As mentioned, the “Angelina Jolie effect” had a significant impact on the awareness of mastectomy but little understanding of the BRCA mutation as determined by surveys [1]. This may relate to our data, which showed a significant spike in the search term “mastectomy”; however, there was very little impact to “BRCA1.” Similarly, in the UK, there was extensive coverage of the diagnosis and death of Jade Goody from cervical cancer and the timing coincided with the release of the HPV vaccine. Jade Goody’s story did have an impact in terms of decisions by women for cervical screening, with a higher impact on women who were young, from lower educational backgrounds and socioeconomic classes [27]. In the period during her diagnosis and death, there were about 500,000 extra cervical screening attendances in England between mid-2008 and mid-2009, and among these, 370 patients had positive results. Furthermore, there were a substantially greater proportion of extra attendances of women aged 25-49 on routine recall in women who were overdue [28-29]. In our study, the highest SVI peak for cervical cancer followed Jane Goody’s death, possibly indicating further awareness, self-education, and, consequently, medical action.

There are several limitations to our study. The principal limitation is that of recall bias, specifically that our search method involved retrospectively searching for an announcement that correlated with a date range, and this could result in an inappropriate correlation between endpoints. While this limitation exists, the common correlations across a variety of cancers with high publicity announcements lend credence to our results.

Notably, these results disproportionately reflect the English-speaking population worldwide based on Internet access, specifically the United States and the United Kingdom. Especially in a worldwide search, this may not be a significant portion of the world population. Moreover, one person could search the term many times, increasing the volume of searches but not necessarily reflecting the overall interest of the population. Furthermore, we can correlate a temporal relationship with available news and a spike in SVI, however, there is no specific way to determine if certain news topics, in fact, triggered the search volume. This would require further surveys to determine the intention or catalyst of the search. Thus, we can only note correlations but no significant relationship. Although Google is the most commonly used search engine, it is not the only one that can be utilized, and it is possible that some cancers are more frequently searched through alternative engines. Finally, there are many spikes that could not be correlated with an event to the best of our knowledge and may require further investigation.

## Conclusions

For many cancer searches, activity and Internet interest are peaked by significant events that occur to famous figures or advances in medicines that are substantially covered in the media. In all cases but “Breast Cancer,” these events portend to higher search activity as compared to campaigns and awareness months. Our study suggests that media coverage of public figures may trigger knowledge-seeking activity. This information demonstrates that public figures announcing and creating a dialog with the media regarding their cancer experience can lead to a more-interested public on major cancer topics. This information can steer future awareness campaign and funding.
